# Influence of women's autonomy on infant mortality in Nepal

**DOI:** 10.1186/1742-4755-8-7

**Published:** 2011-04-19

**Authors:** Ramesh Adhikari, Yothin Sawangdee

**Affiliations:** 1 Geography and Population Department, Mahendra Ratna Campus, Tribhuvan University, Kathmandu, Nepal; 2 Institute for Population and Social Research, Mahidol University, Salaya, Phutthamonthon, Nakhon Pathom 73170, Thailand

## Abstract

**Background:**

Nepalese women lag behind men in many areas, such as educational attainment, participation in decision-making and health service utilization, all of which have an impact on reproductive health outcomes. This paper aims to examine the factors influencing infant mortality, specifically, whether women's autonomy has an impact on infant mortality in the Nepali context.

**Methods:**

Data were drawn from the Nepal Demographic and Health Survey, 2006. The analysis is confined to 5,545 children who were born within the five years preceding the survey. Association between infant mortality and the explanatory variables was assessed using bivariate analysis. Variables were then re-examined in multivariate analysis to assess the net effect of women's autonomy on infant mortality after controlling for other variables.

**Results:**

The infant mortality rate (IMR) in the five years preceding the survey was 48 deaths per one thousand live births. Infant mortality rate was high among illiterate women (56 per 1000 live births) and among those not involved in decision making for health care (54 per 1000 live births). Furthermore, infant mortality was high among those women who had more children than their comparison group, who had birth intervals of less than two years, who had multiple births, who were from rural areas, who were poor, whose source of water was the river or unprotected sources, and who did not have a toilet facility in their household.

Results from logistic regression show that women's autonomy plays a major role in infant mortality after controlling other variables, such as mother's sociodemographic characteristics, children's characteristics and other household characteristics. Children from literate women had a 32 percent lower chance (OR = 0.68) of experiencing infant mortality than did children from illiterate women. Furthermore, infants of women who were involved in decision-making regarding their own health care had a 25 percent lower (OR = 0.75) chance of dying than did infants whose mothers who were not involved in healthcare decisions.

**Conclusion:**

Infant mortality is high in Nepal. In this context, mother's literacy and involvement in healthcare decision making appear to be the most powerful predictors for reducing infant mortality. Hence, in order to reduce infant mortality further, ongoing female education should be sustained and expanded to include all women so that the millennium development goals for the year 2015 can be attained. In addition, programs should focus on increasing women's autonomy so that infant mortality will decrease and the overall well being of the family can be maintained and enhanced.

## Background

As in other many developing countries, high infant mortality has been a major public health problem in Nepal. Although over the past few decades, Nepal has seen substantial improvements in its reproductive health outcomes, infant and child mortality as well as maternal mortality remain high compared to rates in other developing countries.

In many societies women's inferior social status and status within the household adversely affect their health and that of their children. The health of women and their children is largely impaired by culturally and socially determined roles for women through a complex web of physiological and behavioral interrelationships and synergies that permeate every aspect of their lives [1]. Much research has shown that higher status for women correlates positively with their and their children's health [2,3].

It is well documented that women almost everywhere are disadvantaged compared to men in terms of their access to assets, employment, health care, and education. Since the social status and level of autonomy of Nepali women is low, their status at the household level needs to be further explored in terms of health services utilization, which has a direct impact on maternal and infant morbidity and mortality. This relationship clearly warrants further attention, particularly in settings such as Nepal, where maternal and child health utilization is low [4].

A substantial body of research has examined the role of women's autonomy on health and behavioral outcomes such as fertility [5,6], family planning [7-9], and child rearing and pregnancy care [10-13]. However, only a few studies have assessed the role of women's autonomy on child survival or infant mortality [14,15].

This study is an attempt to examine the prevalence and factors influencing infant mortality. More specifically, it aims to investigate whether women's autonomy has an impact on infant mortality in Nepal. In addition, this paper also aims to close the knowledge gap in the literature with regard to a society in which women suffer gross disadvantages in the context of a patriarchal culture, which in turn can help guide reproductive health program planners and policy makers to understand various factors influencing infant mortality and to assist in implementation of reproductive health programs that will decrease infant morbidity and mortality. Though there are a few studies that have assessed the relationship between women's autonomy and infant mortality outside the country, to our knowledge this type of research has not yet been undertaken in Nepal.

## Data and Methods

This paper reports on data drawn from the Nepal Demographic and Health Survey (NDHS), 2006, a nationally representative sample survey. The primary purpose of the 2006 NDHS was to furnish policymakers and planners with detailed information on fertility, family planning, infant/child/adult/maternal mortality, maternal and child health, nutrition, and knowledge of HIV/AIDS and other sexually transmitted infections. The 2006 NDHS was carried out under the aegis of the Population Division of the Ministry of Health and Population. The data from this survey is openly available.

The 2006 NDHS used the sampling frame provided by the list of census enumeration areas with population and household information from the 2001 Population Census. Each of the 75 districts in Nepal is subdivided into Village Development Committees (VDCs), and each VDC into wards. The primary sampling unit (PSU) for the 2006 NDHS is a ward, sub-ward, or group of wards in rural areas, and sub-wards in urban areas. The sample for the survey is based on a two-stage, stratified, nationally representative sample of households. At the first stage of sampling, 260 PSUs (82 in urban areas and 178 in rural areas) were selected using systematic sampling with probability proportional to size. At the second stage of sampling, systematic samples of about 30 households per PSU on average in urban areas and about 36 households per PSU on average in rural areas were selected in all the regions. Interviews were completed for 10,793 women of reproductive age.

Infant mortality rate (IMR) is defined as the number of deaths of infants under age 1 per 1,000 live births in a given year. In the present study, women's autonomy refers to the education status of women, and women's household decision-making autonomy, which was measured based on responses to "Who makes the following decisions in (respondent's) household about: 1) obtaining health care for yourself; 2) large household purchases; 3) household purchases for daily needs; and 4) visits to family or relatives?" Response options were: a) respondent alone; b) respondent and husband/partner; c) respondent and other person; d) husband/partner alone; e) someone else; f) other. The value of 1 is assigned if the response was (a), (b), or (c), that is, involvement of the respondent, or else 0, for no involvement of the respondent. The other independent variables included in this study were demographic and socioeconomic variables such as age, religion, number of children born, previous birth interval, sex of the child, type of birth, place of residence, ecological zone, wealth status of households, sources of drinking water, and availability of toilet facilities.

The unit of analysis in our study is children who were born within the five-year period (n = 5545) preceding the survey interview. Data were weighted to represent the structure of Nepali population using weighting factors provided with the NDHS. Association between infant mortality and the explanatory variables was assessed in bivariate analysis using Chi-square tests. Logistic regression was used to assess the net effect of mother's autonomy on infant mortality after controlling several other control variables. Two models were used in the analysis. The first model contained the variables related to women's autonomy. In the second model, other demographic and socioeconomic characteristics were added. Prior to the multivariate analysis, multi-collinearity between the variables was assessed. However, no multi-collinearity was found among the variables. Only those variables that were significant in the bivariate analysis were further analyzed in logistic regression. The Statistical Package for Social Science (SPSS 17.0 for Windows) software was used to analyze the data.

## Results

Three out of five mothers (60%) were illiterate. Only about half or less (43%-51%) of mothers were involved in decisions regarding their own health care, large household purchases, household purchases for daily needs, and visits to family or relatives. More than two-fifths of mothers (42%) were below 25 years of age. A considerable proportion of the infants (32%) had four or more siblings. A majority of the mothers had birth spacing of two-three years. More than a quarter of households drank water from unprotected sources (e.g., a river or unprotected well). Notably, more than three-fifths (63%) of houses did not have toilet facilities at the time of the survey (Table 1).

**Table 1:** Selected background characteristics

Characteristics		%	N
**Women's autonomy**			

**Literacy status**	Illiterate	60.3	3,343
	Literate	39.7	2,202

**Decision on own health care**	Without involvement of respondent	56.7	3,146
	Involvement of respondent	43.3	2,398

**Decision on making large household purchases**	Without involvement of respondent	55.0	3,047
	Involvement of respondent	45.0	2,497

**Decision on making household purchases for daily needs**	Without involvement of respondent	49.3	2,734
	Involvement of respondent	50.7	2,811

**Decision on visits to family or relatives**	Without involvement of respondent	50.6	2,808
	
	Involvement of respondent	49.4	2,736

**Demographic and socioeconomic characteristics**			

**Age group**	<25	41.5	2,299
	25-29	31.4	1,742
	30 or older	27.1	1,504

**Religion**	Buddhist	7.4	411
	Hindu	84.9	4,706
	Muslim/other	7.7	428

**Total children ever born**	One	19.7	1,094
	Two	29.7	1,649
	Three	18.5	1,025
	Four or more	32.1	1,777

**Previous birth interval**	<2 years	21.9	847
	2-3 years	55.6	2,148
	4+ years	22.4	866

**Source of drinking water**	Piped/tube well water	72.4	4,014
	River/stone tap/unprotected well	27.6	1,531

**Toilet facility**	Yes	37.2	2,061
	No facility/bush/field	62.8	3,484

**Total**		**100.0**	**5,545**

The infant mortality rate in the five years preceding the survey was 48 deaths per one thousand live births. The IMR varied significantly with different settings. For instance, the IMR was higher among illiterate women (56 per 1000 live births) than among literate women (35 per 1000 live births). Similarly, the IMR was significantly higher among women who were not involved in decision making for their own health care (54 per 1000 live births) than among those who were involved in the decision-making process for their own health care (40 per 1000 live births). Furthermore, the IMR was lower among women aged 25-29 (39 per 1000 live births) than among other age groups (56 per 1000 live births among women aged 15-24 and 46 per 1000 live births among women aged 30 or above). Moreover, the IMR was significantly higher among Muslim women, who had more children (36 per 1000 live births among women who had one child and 59 per 1000 live births among those who had four or more children). Similarly IMR was significantly higher among those who had less than two-year birth intervals (62 per 1000 live births), among those who had multiple births (382 per 1000 live births), among those who lived in the Mountain region (75 per 1000 live births), among those who were poor (54 per 1000 live births), among those whose sources of drinking water was a river or other unprotected sources (57 per 1000 live births), and among those who did not have toilet facilities in their house (53 per 1000 live births) than their comparison group (Table 2).

**Table 2:** Infant mortality rates (per 1,000 live births) for 5-year periods preceding the survey, by background characteristics (N = 5,545)

Characteristics		IMR
**Women's Autonomy**		

Literacy status ***	Illiterate	56
	Literate	35

Decision on own health care *	Without involvement of respondent	54
	Involvement of respondent	40

**Decision on making large household purchases**	Without involvement of respondent	50
	Involvement of respondent	45

**Decision on making household purchases for daily needs**	Without involvement of respondent	53
	Involvement of respondent	45

**Decision on visits to family or relatives**	Without involvement of respondent	53
	Involvement of respondent	42

**Sociodemographic variables**		

Age group *	<25 years	56
	25-29	39
	30 or more	46

Religion *	Buddhist	26
	Hindu	48
	Muslim/other	65

Total children ever born *	One	36
	Two	43
	Three	48
	Four or more	59

Previous birth interval **	<2 years	62
	2-3 years	41
	4+ years	27

**Sex of child**	Female	53
	Male	42

Type of births ***	Singleton	42
	Multiple	382

**Place of residence**	Urban	34
	Rural	50

Ecological zone ***	Terai	53
	Hill	36
	Mountain	75

Wealth status **	Poor/middle	54
	Rich	35

Source of drinking water *	Piped/tube well water	44
	River/stone tap/unprotected well	57

Availability of Toilet facility*	Yes	39
	No facility/bush/field	53

**Total**		48

Note *** Significant at p < 0.001; ** = p < 0.01 and * = p < 0.05

In the first model of logistic regression, both women's autonomy-related variables had a statistically significant effect on infant mortality. Literate women had 39 percent lower odds (OR = 0.61) of experiencing infant mortality than did illiterate women. Similarly, women who were involved in the decision-making process for their own health care had a 26 percent lower chance (OR = 0.74) of experiencing infant mortality than those who were not involved in the decision-making process regarding their own health care.

Model 2 presents the final results after adding other demographic and socioeconomic characteristics of the women, as well as household characteristics. Even after inclusion of these variables in model 2, women's autonomy variables retained their significance level. Furthermore, the reduction of the significance level of women's autonomy variables after inclusion of the other variables indicates that demographic and socioeconomic variables are also important predictors for infant mortality. Moreover, women's age, number of children ever born, religion, previous birth interval, and type of births were also significant predictors of infant mortality. Age has a negative and statistically significant impact on infant mortality; as the age of women increased, infant mortality decreased. On the other hand, the larger the number of a woman's total number of children ever born the larger the IMR. Infants born of Hindu and Muslim women were more likely to die than those born of Buddhist women. Furthermore, infants from mothers who had two-or-three-year and four-year or more birth intervals had 55 percent and 68 percent, respectively, lower odds of dying than did infants whose mothers had previous birth intervals of less than two years. Notably, infants born as twins were more likely (OR = 14.5) to die before reaching one year than were single-birth infants (Table 3).

**Table 3:** Adjusted odds ratios (OR) and 95% confidence interval (CI) for infant mortality within the past five years preceding the survey by selected predictors

Selected predicators		Model I		Model II	
		
		OR	CI	OR	CI
**Women's Autonomy**					

**Literacy status**	Illiterate (ref.)	1.00		1.00	
	Literate	0.61***	0.47-0.80	0.65*	0.47-0.90

**Decision on own health care**	Without involvement of respondent (ref.)	1.00		1.00	
	Involvement of respondent	0.74*	0.57-0.95	0.75*	0.57-0.98

**Sociodemographic variables**					

**Age**	<25 years (ref.)			1.00	
	25-29			0.57**	0.39-0.83
	30 or more			0.57**	0.36-0.91

**Total number of children ever born **	One (ref.)			1.00	
	Two			1.62*	1.07-2.45
	Three			2.58***	1.59-4.19
	Four or more			3.21***	1.89-5.45

**Religion **	Buddhist (ref.)			1.00	
	Hindu			1.96*	1.02-3.79
	Muslim/other			2.32*	1.08-5.02

**Previous birth interval **	<2 years (ref.)			1.00	
	2-3 years			0.45***	0.33-0.61
	4+ years			0.30***	0.18-0.50

**Type of births **	Singleton (ref.)			1.00	
	Multiple			14.5***	8.89-23.6

**Ecological zone **	Terai (ref.)			1.00	
	Hill			0.81	0.58-1.12
	Mountain			1.47	0.97-2.25

**Wealth status **	Poor/middle (ref.)			1.00	
	Rich			0.83	0.59-1.17

**Source of drinking water **	Piped/tube well water (ref.)			1.00	
	River/stone tap/unprotected well			1.24	0.91-1.68

**Availability of Toilet facility**	Yes (ref.)			1.00	
	No facility/bush/field			0.82	0.59-1.14

**Constant**		0.07***		0.04***	
**-2 Log likelihood**		2105.6		1918.5	
**Cox & Snell R Square**		0.003		0.037	

Note *** Significant at p < 0.001; ** = p < 0.01 and * = p < 0.05

## Discussion

This study aimed to examine the factors influencing infant mortality in Nepal, specifically, by investigating whether the women's autonomy has an impact on infant mortality. Results show that infant mortality is high in Nepal, thus indicating an unmet need to be addressed by maternal and child health programs.

Bivariate analysis shows that variables such as women's autonomy (namely, literacy status of women and decision making regarding their own health care), religion, number of children ever born, previous birth interval, type of birth (single or multiple), ecological zone, wealth status, sources of drinking water, and availability of toilet facilities in a household are important variables in explaining infant mortality. The multivariate analysis supported most of the findings of the bivariate analysis. In the multivariate analysis women's autonomy (literacy status and decision making regarding their own health care), age group, total number of children born, previous birth interval, and type of birth were significant predictors of infant mortality.

As do many other studies, this study also shows that literate mothers have a lower experience of infant deaths. It could be that educated mothers are more capable of accessing available health facilities and that they are able to greatly change the traditional balances of power and autonomy in familial relationships, with profound effects on child care [16]. Moreover, education can contribute to children's survival by making women more likely to marry and give birth later and to have fewer children, utilize prenatal care, and immunize their children [17]. Anther reason could be that schools are institutions that transform young girls into empowered, assertive, and confident women [18].

Our study showed that infant mortality is significantly lower among those mothers who were involved in decisions regarding their own health care compared to those who were not. A possible explanation could be that women who have autonomy in decision making are more likely to have a higher level of contraceptive use, which might lessen their reproductive behavior risks, prolong birth intervals, lower fertility [5], and result in lower infant mortality. A study in India has confirmed that a women's control over household resources (ability to keep money aside) has a significant positive effect on both the demand for prenatal care and the probability of hospital delivery [19].

Our study showed that infants from older women were less likey to die before reaching their first birthday. It could be that the bodies of young mothers are not selective enough with respect to malformed fetuses, which results in higher rates of stillbirths and hypothrophic births, and thus in higher infant mortality [20-22]. As do many other studies, this study shows a strong relationship between fertility behavior of women and child death. Infants born of those women who had given birth to more children were more likely to die than were infant froms those mothers who had given birth to only one child. It could be that as a family grows the parental resources might be insufficient to maintain the same level of nutrition for a larger number of children, and thus those born earlier might enjoy better nutritional status than those born later [23]. Short birth spacing and high birth order may affect both maternal and fetal health as well as availability of time for child care. This finding conforms with other studies that find that the length of the birth interval is positively correlated with the survival of the infant child [24-27]. Our study found that the probability of infant mortality is very high in the case of multiple births. This effect is mainly associated with the lower birth weight of twins or triplets, which in trun is one of the most important factors affecting neo-natal survival. The reason could be that the arrival of more than one child also creates extra demand for food. During the early stages of infancy, since breast feeding is one of the main sources of nutrition, multiple births might lead to infants' lower calorie intake, and thus to lower survival chances [28].

This study found that infants born of Hindu and Muslim women were more likley to die than were infants from Buddhist women. It could be that Hindu and Muslim girls tend to marry at an earlier age, which leads to conception at an earlier age, which in turn increases the risk of child morbidity and mortality. The other reason could be that cultural beliefs and practices often lead to self-care, home remedies, and consulation with traditional healers [29]. These factors might result in delays in treatment seeking, which are more common among women, not only with regard to their own health but, especially, with regard to children's illnesses [30-32].

There are some limitations in the interpretation of the results of this study. Women's autonomy is a complex phenomenon that cannot be completely measured by education and only a few household decision-making indicators. Similarly, as pointed out previously, we restricted our subjects to mothers whose infants who were born within the five years preceding the survey, so our results regarding the prevalence of infant mortality should be generalized with care. Comparisons between surveys should also be interpreted with caution because quality of data and sample coverage varies. Because the cross-sectional design of the study and all of the items analyzed in the logistic regression analysis came from information at the time of survey, the analysis can only provide evidence of statistical association between those items and infant mortality and cannot show cause-effect relationships. Furthermore, we should not forget other important errors that could exist in the collection of information regarding infant deaths. Women tend to omit noting children who may have died in very early days after their birth. However, since the 1991 fertility and family planning survey in Nepal, it has been argued that the quality of pregnancy history data has improved and that there is very little omission of births and deaths, especially during the recent past. As the effect of these omissions on the calculation of demographic rates is minimal, a direct method of estimation has been used since then [33]. There may in fact be very few errors in this regard as it is believed that the Nepal Demographic and Health Survey data were good enough to estimate mortality directly [4].

## Conclusions

In Nepal, women's autonomy is a strong predictor among many other predictors of infant mortality. Mothers' literacy and decision-making power regarding health care appear to be the most powerful predictors among many others for reducing infant mortality. Hence, in order to reduce infant mortality further, ongoing female education should be sustained and broadened to include every woman in order to reach the MDG goal for the year 2015. If programs focus on increasing women's autonomy, infant mortality will decrease and the overall well being of the family will be maintained and be enhanced.

## Competing interests

The authors declare that they have no competing interests.

## Authors' contributions

RA analyzed and interpreted the data and drafted the manuscript. YS commented on the analysis and provided suggestions. Both authors read and approved the final version of the manuscript.
